# Supplementary material for: Causal Relationship between Aging and Anorexia Nervosa: A White-Matter-Microstructure-Mediated Mendelian Randomization Analysis
Source: Biomedicines. 2024 Aug 16;12(8):1874. doi: 10.3390/biomedicines12081874 (PMC11351342; doi:10.3390/biomedicines12081874)
Supplement: Supplementary file 1 [file biomedicines-12-01874-s001.zip › Supplementary Figure/Figure S8/Leaveoneout plot of MR analysis for TL to ubm-b-1858.pdf]

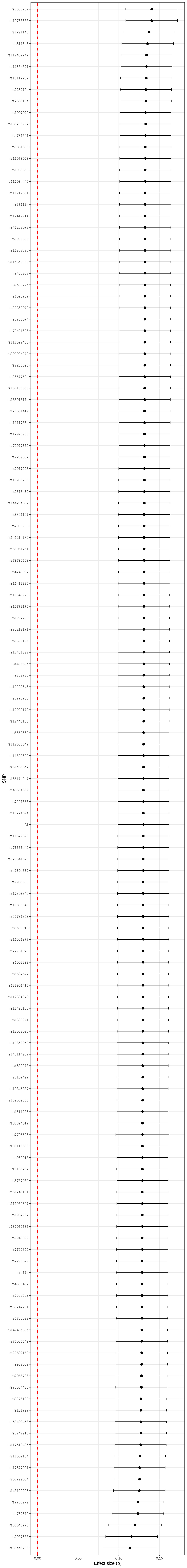
SNP
rs6536702
rs10768683
rs1291143
rs611646
rs117407747
rs11584821
rs10112752
rs2282764
rs2555104
rs6007020
rs139795227
rs4731541
rs6881568
rs16978028
rs1985369
rs117034449
rs11212631
rs871134
rs12412214
rs41269079
rs3093888
rs11769630
rs116863223
rs450962
rs2538745
rs1023767
rs28363070
rs3785074
rs78491606
rs111527438
rs202034370
rs2230590
rs28577594
rs150150565
rs188918174
rs73581419
rs11117354
rs12925933
rs79977579
rs7209057
rs2977608
rs10905255
rs9878436
rs144204502
rs3891167
rs7099229
rs141214782
rs56061761
rs73730598
rs4743037
rs11412296
rs10840270
rs10773176
rs1907702
rs76219171
rs9398196
rs12451892
rs4498805
rs869785
rs13230646
rs6776756
rs12932179
rs17445108
rs6659669
rs117630647
rs11699829
rs61405042
rs185174247
rs45604339
rs7221585
rs10774624
All
rs11579626
rs76666449
rs376641875
rs41304832
rs9955360
rs17803849
rs10805346
rs66731853
rs9600019
rs11991877
rs77231040
rs1003322
rs6587577
rs137901416
rs112394943
rs11426156
rs1332941
rs13062095
rs12369950
rs145114957
rs4530278
rs8102497
rs10845387
rs139669835
rs1611236
rs80324517
rs7705526
rs80116508
rs939916
rs8105767
rs3767952
rs61748181
rs111950327
rs1957937
rs182059586
rs9940099
rs7790856
rs2293579
rs4724
rs4695407
rs6669563
rs55747751
rs6790988
rs142426306
rs76065543
rs28502153
rs932002
rs2056726
rs75664430
rs2276182
rs131797
rs59409453
rs5742915
rs117512405
rs11557154
rs17677991
rs56799554
rs143190905
rs2763979
rs762679
rs35640778
rs2967355
rs35446936
0.00
0.05
0.10
0.15
Effect size (b)
